# Wireless muometric navigation system

**DOI:** 10.1038/s41598-022-13280-4

**Published:** 2022-06-16

**Authors:** Hiroyuki K. M. Tanaka

**Affiliations:** University of Tokyo, Tokyo, Japan

**Keywords:** Physical oceanography, Geophysics, Experimental particle physics, Electrical and electronic engineering, Particle astrophysics

## Abstract

While satellite-based global navigation systems have become essential tools in our daily lives, their effectiveness is often hampered by the fact that the signals cannot be accessed in underground, indoor, or underwater environments. Recently, a novel navigation system has been invented to address this issue by utilizing the characteristics of the ubiquitous and highly penetrative cosmic-ray muons. This technique, muometric navigation, does not require active signal generation and enables positioning in the aforementioned environments within a reference coordinate defined by the three-dimensional positions of multiple detectors. In its first phase of development, these reference detectors had to be connected to the receivers via a wired configuration to guarantee precise time synchronization. This work describes more versatile, wireless muometric navigation system (MuWNS), which was designed in conjunction with a cost-effective, crystal-oscillator-based grandmaster clock and a performance evaluation is reported for shallow underground/indoor, deep underground and undersea environments. It was confirmed that MuWNS offers a navigation quality almost equivalent to aboveground GPS-based handheld navigation by determining the distance between the reference frame and the receivers within a precision range between 1 and 10 m.

## Introduction

In recent decades, Global Positioning System (GPS) or Global Navigation satellite system (GNSS) has become increasingly prominent, since transportation (aircrafts, boats, cars, and even excursions on foot) frequently relies on GPS for navigation and tracking, often from handheld devices. Wireless navigation, using a combination of GPS and portable receivers embedded in aircraft/boat/car navigation systems or smartphones, has supported transportation or directional orientation^1–4^, checking patterns of human movement^5–7^, and even health tracking^8–10^. However, the range of GPS does not extend to underground/indoor and underwater environments, therefore in those regions, navigation capability is still quite limited. Currently, the most common methods to use for navigation in such environments are based on acoustic^11^, Wi-Fi^12^, cellphone signals^13^, Bluetooth low energy (e.g., iBeacon), Indoor messaging system (IMES)^14^, and ultrasonic techniques^15^. However, all of these systems need an active source; hence they require pre-installed infrastructures such as acoustic transmitters/transceivers, Wi-Fi access points and/or cell towers and/or iBeacon/IMES transmitters, and generally these high-cost resources are only available in highly populated areas. When these infrastructures are not available or only partially available, the positioning accuracy will be low. For example, although the cellphone-based navigation technique can be used for navigation in mountainous and underground environments as long as the cellphone signals are available, the positioning accuracy is limited to a few km to a few tens of kilometers^16^. On the other hand, the pedestrian dead reckoning (PDR)^17^ and barometric^18^ techniques are passive techniques and thus, these techniques don't require pre-installed infrastructure developments; however, since these techniques only measure the relative positions or can only determine the elevation information, generally these techniques have to be combined with other active techniques.

Recently, a novel passive navigation system using cosmic rays was invented^19^ as an underground and underwater navigation system, with the objective that it could also be applied to navigation at high latitudes where GPS works poorly^20^. The technique is called the muometric navigation system or muometric positioning system (muPS) and utilizes the ubiquitous and penetrative nature of cosmic ray muons; these particles have also been applied as probes for muography to visualize interior structures of gigantic objects such as volcanoes and pyramids^19–32^. When these probes, which are naturally occurring, are adapted for navigation, the receiver detector's position can be determined with centimeter-level accuracy within the coordinates defined by the reference detectors.

**Figure 1:**
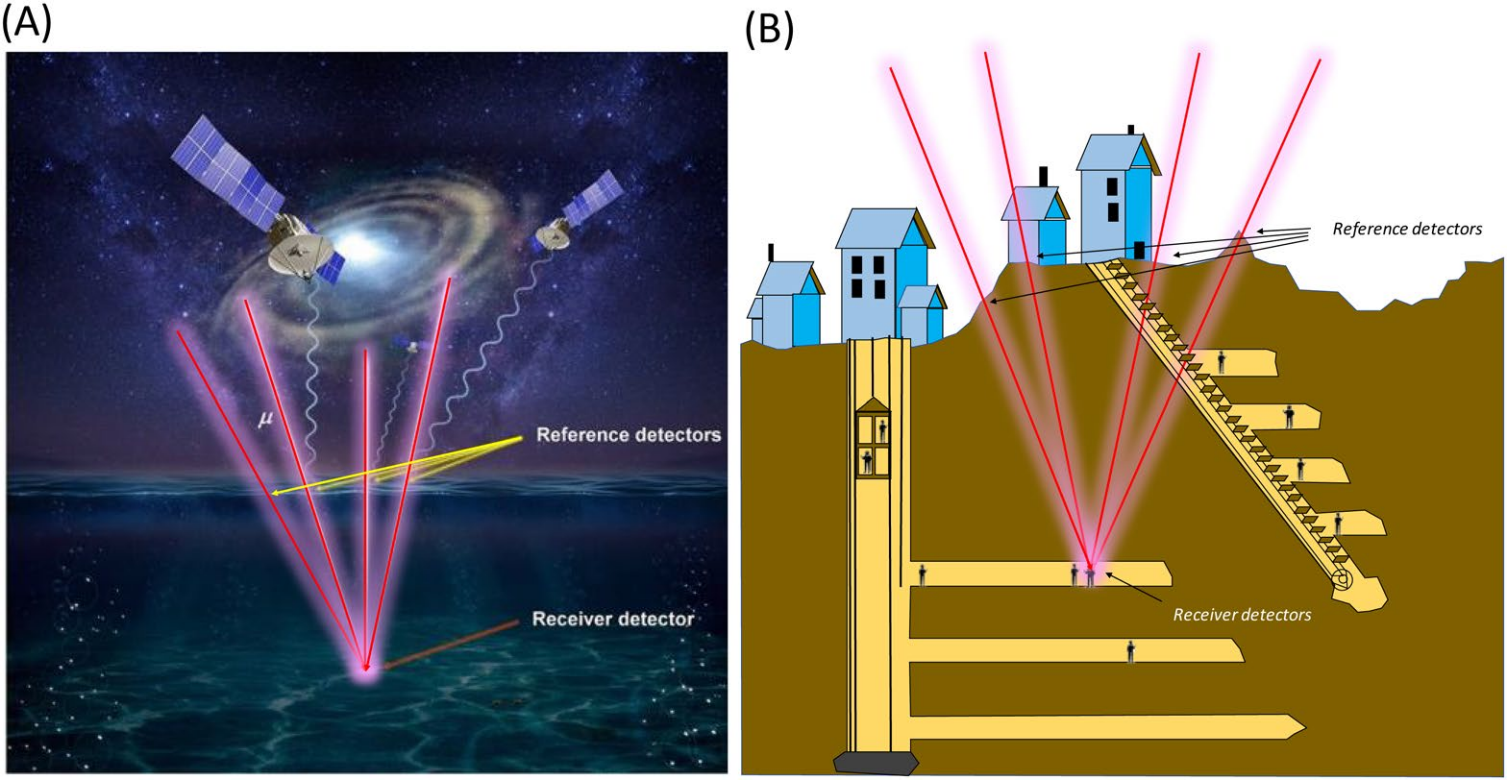
Principle of muPS. For underwater navigation, multiple reference detectors are located on the sea surface to navigate underwater receivers (**A**), and for underground navigation, multiple reference detectors are located on the ground surface to navigate underground receiver (**B**). The red lines indicate the muon trajectories. The copyright for these images is owned by HKMT.

The principle of muPS is similar to that of GPS. The muPS technique requires, instead of satellites, three or more reference detectors to form the reference coordinate (Fig. 1). The receiver detector defines the relative position within this coordinate by using the following relationship:

1 $$ L_{i}^{2} = \left( {x_{i} - x_{{\text{p}}} } \right)^{2} + \left( {y_{i} - y_{{\text{p}}} } \right)^{2} + \left( {z_{i} - z_{{\text{p}}} } \right)^{2} , $$

where *L*_*i*_ is the geometrical distance between the *i*th reference detector located at (*x*_*i*_*, y*_*i*_*, z*_*i*_) and the receiver detector located at (*x*_p_, *y*_p_, *z*_p_). Since *L*_*i*_, *x*_*i*_*, y*_*i*_*,* and *z*_*i*_ are known, Eq. (1) has three variables; hence a ternary operation of Eq. (1) with three independent muon tracks will suffice. As can be seen in the reference^19^, in the operation of wired muPS introduced in the prior work, since the reference detectors and the receiver detectors are connected with physical cables, the reference and receiver detectors are perfectly synchronized (also there is no drift effect); hence the time offset due to non-synchronicity doesn’t exist between these detectors. Therefore, the number of parameters to be derived is only three (*x*_p_, *y*_p_, *z*_p_) and thus, unlike GPS, three is the minimum number of the reference detectors required for positioning. Since the muon's traveling speed is approximately the speed of light in a vacuum (*c*_0_)^19^, the distance (*L*_*i*_) between the reference detector and the receiver detector is determined by the muon's time of flight (TOF) between them.

For a given distance between the reference and the receiver detectors, the TOF spectrum has a standard deviation (SD) (*σ*_TOF_) of 1 ns with detectors that consist of plastic scintillators and photomultipliers (PMTs)^19^, where the size of the scintillator is 20 × 20 cm^2^. This jitter comes from the rise time of scintillators, PMTs and signal discriminations with constant fraction discriminators (CFDs). The jitter coming from the time to digital converter (TDC) increases as the measurement time range becomes longer, and they were 50 ps, 200 ps, and 700 ps (standard deviation) for the time intervals of 50 ns, 500 ns, and 5 μs, respectively^19^. In the prior work, the positioning process was repeated 7 times for confirmation of reproducibility and for estimating the positioning errors (standard deviation) associated with the x, y, and z directions, and it was confirmed that a-few-centimeter level accuracy could be achieved for a few m base line length^19^. By averaging over multiple muon tracks, this spectrum will be naturally sharpened. According to the reference^19^, the relationship between the number of muon tracks (*N*_µ_) and this jitter (*σ*_TOF_) is expressed by:

2 $$ (N_{\upmu } )^{{ - {1}/{2}}} \sim k\sigma_{{{\text{TOF}}}} \left[ {{\text{ps}}} \right], $$

where the coefficient *k* = 6 × 10^2^.

As aforementioned in the prior work^19^, the first muPS experiment operated via a cable, which conveyed power supply and data connections to the reference detectors, making it possible to manage the system reliably in terms of time synchronicity. However, the cable inherently limits the mobility of the receiver detectors due to cable strain and entanglement risks. When the cables connecting reference detectors with the receiver detector are removed, muPS is more flexible and thus adaptable to a wider-range of applications. MuWNS would help to avoid such issues by removing the need for a physical cable. This new capability would be useful in several cases. For example, interest has recently increased in the search for a solution for better navigation of remote controlled underwater autonomous systems called Remotely Operated Vehicles (ROVs), which are used for monitoring the underwater environment^33^. They are now controlled through umbilicals (physical cables) which limits the mobility of ROVs. However, in order to deploy a wireless underwater vehicle, the interference between the acoustic communication and the acoustic positioning systems must be avoided^34^. Moreover, the speed of sound in water varies by more than 5% (from 1426.5 to 1507 ms^−1^) as the water temperature varies from 5 to 30 °C, and can result in a large positioning error where there is a large uncertainty in water temperatures (> 50 m for a 1 km baseline at an uncertainty level of 25 °C). The current work offers our view on the feasibility of wireless muPS operations that could be designed not to interfere with acoustic communications. Wireless muPS could be achieved by combining the long-range time-to-digital converter (TDC) and the grandmaster clock's (GMC's) "holdover"^35^ mode with oven-controlled crystal oscillators (OCXOs). The design of this new wireless muPS that works in conjunction with this OCXO-based GMC's holdover will be presented and its performance will be evaluated.

## Results

### Principle of MuWNS

During operation of MuWNS, the time synchronization between the reference clocks and receiver clocks is performed by using a grandmaster clock's (GMC's) "holdover"^35^ mode with oven-cooked crystal oscillators (OCXOs). Although GPS signals are one of the most common time synchronization signal inputs, this solution does not function in indoor, underground or underwater environments since GPS signals are not available in such environments. The GMC's "holdover"^35^ mode functions reliably for a certain duration when the GPS synchronization input has been disrupted or temporarily unavailable. Although OCXOs are one of the standard clocks available which provide reliable holdover measurements, they have intrinsic frequency-drift, causing a time offset error (Δ*t*) between the reference clocks and receiver clocks. Therefore, for MuWNS, Eq. (1) will have one more variable such that:

3 $$ L_{i}^{{2}} = \left( {x_{i} - x_{{\text{p}}} } \right)^{{2}} + \left( {y_{i} - y_{{\text{p}}} } \right)^{{2}} + \left( {z_{i} - z_{{\text{p}}} } \right)^{{2}} + s^{{2}} , $$

where *s* (= *c*_0_Δ*t*) is the pseudo-length that comes from the time offset at the receiver detector. Consequently, during operation of MuWNS, at least four reference detectors are needed for positioning. Therefore, the principle of MuWNS is closer to the principle of GPS that requires at least four satellites for positioning.

However, this scheme works only when we can reasonably assume that the time offset (Δ*t*) stays the same within the time range required for positioning (the time to collect at least four muon tracks at each pair of the reference and the receiver detectors), which depends on the rate of the muon that passes the reference and the receiver detectors. This means on the other hand, as long as we can approximate this Δ*t* is constant, the method can be used in the environment where GPS signals cannot be received for a long time. In this paper, based on the measurement results of the OCXO’s drift level and the open-sky muon energy spectrum, the relationship between this time offset, the detector size, and the distance between the reference and the receiver detectors will be discussed and optimized for three possible scenarios of MuWNS operations. For simplicity, it was assumed that the reference detectors were constantly connected to the GPS antenna and getting an uninterrupted signal.

### Available muon rate

The muon's tracking frequency (*N*^−1^) (the time required to collect a muon event that passes both the reference and the receiver detectors) can be derived by integrating the open-sky zenith angular muon's energy spectrum *I* (*E*, *θ*)^36–38^ over the energy range between *E*_c_ and infinity:

4 $$ N(q) = \mathop \int \limits_{{E_{c} }}^{\infty } I\left( {E,\theta } \right)dE, $$

where *E*, *θ*, *ϕ* are respectively the muon energy, elevation angle, and azimuth angle. *E*_c_ is the muon cutoff energy that depends on azimuthal variations of matter thickness located above the detector. For a derivation of *E*_c_, the muon's constant slowing down approximation (CSDA)^39^ can be used. In order to keep the relativistic energy of muons in a state sufficiently higher than their invariant mass, *E*_c_ in Eq. (4) was set to be 1 GeV higher than the cutoff energy directly derived from the CSDA muon range. *N* in Eq. (4) is also a function of the solid angle (*Ω*) formed by the reference and receiver detectors. If the size of the detectors (*S*) and the distance (*D*) between the reference and the receiver detectors are given, the solid angle (*Ω*) is approximated as:

5 $$ \Omega \sim S^{2} D^{ - 2} \,{\text{for}}\,S^{2} \, < < \,D^{2} . $$

### Required accuracy for MuWNS

In order to evaluate what the most practical positioning precision will be for each given situation, referencing the accuracy of commonly used portable GPS receivers is useful since the studies reporting the accuracy of these kinds of GPS receivers are extensive. For example, von Watzdorf and Michahelles^40^ reported that the average accuracy of location information ranged between 108 and 655 m with GPS enabled Apple iPhones, iPods, and iPads. Zandbergen^41^ reported that the average horizontal position error of an iPhone was around 10 m, and Garnett and Stewart^42^ found better precision for positioning with an iPhone 4S which was around 6.5 m. However, these varieties of the positioning accuracy depend on the environment (such as how dense the buildings and trees, or topography are) since large structures surrounding the GPS receiver can cause multipath errors. In this work, we define the following three positioning precision categories for practical muometric navigation: Very Precise Level (VPL), a precision better than 1 m (Δ*L*_*i*_ ≤ 1 m); Precise Level (PL), a precision better than 3 m (Δ*L*_*i*_ ≤ 3 m); and Usable Level (UL) a precision better than 10 m (Δ*L*_*i*_ ≤ 10 m). Positioning accuracy worse than UL will be defined as an unpractical level and will not be discussed in this work.

### Design of MuWNS

**Figure 2:**
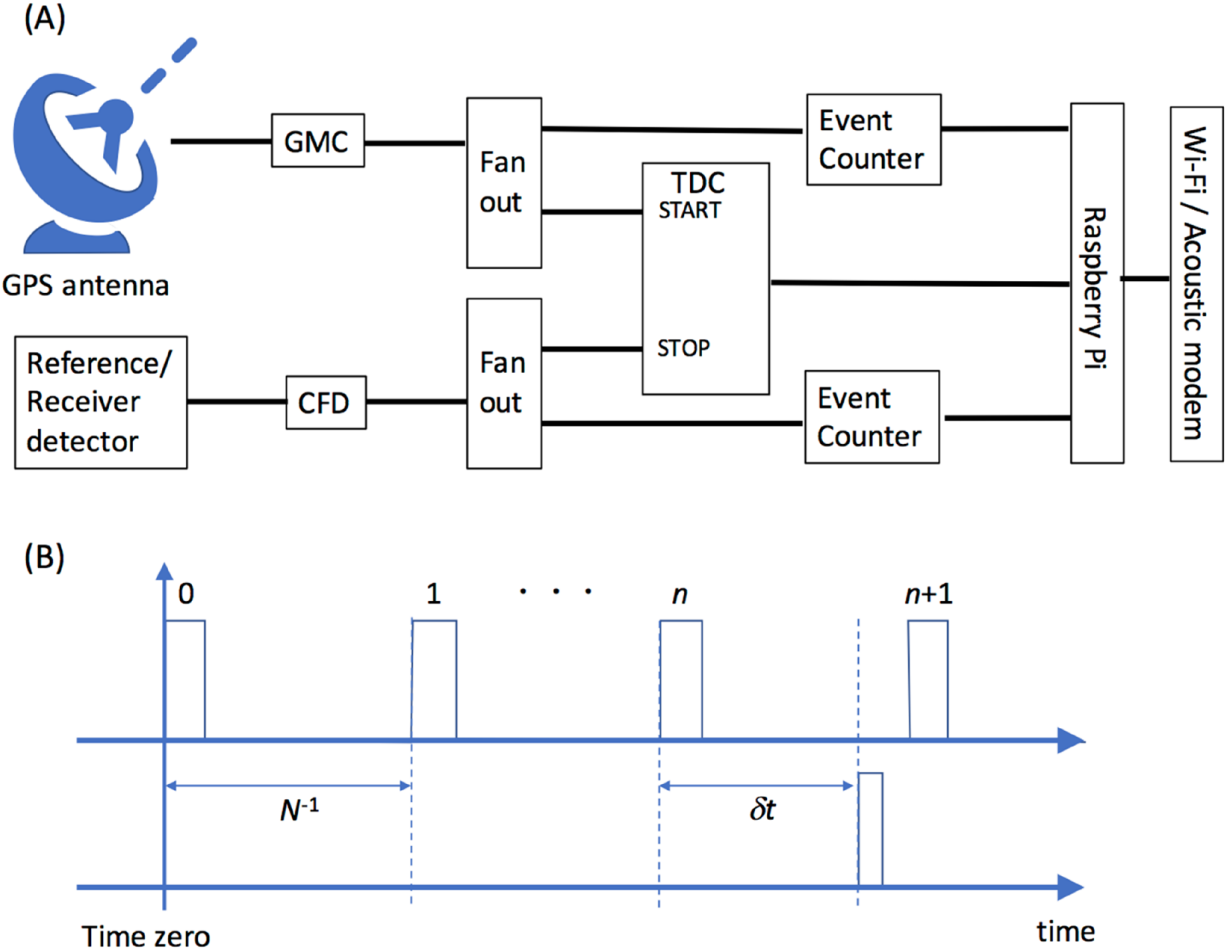
Block diagram of the current wireless muometric positioning/navigation system (MuWMS) (**A**). Both reference detectors and receiver detectors are initially connected with a GPS antenna, but the receiver detectors will be eventually disconnected from the GPS signals when entering indoor/underground/underwater regions. The time series of the logic levels output from the GMC (top) and those output from the TDC (bottom) are illustrated in (**B**). "Time zero" indicates the moment the reference signal is sent to the event counters. *N*^−1^ is the GMC signal output period that usually ranges from 10 to 100 ns. *δt* is the time difference between the GMC signal pulse input and the TDC signal pulse input.

Figure 2 shows the block diagram of the current MuWNS. The time of the MuWNS setup is defined by the GMC, and every MuWNS detector records absolute time information. The GMC is connected to the GPS antenna when GPS signals are available. When GPS signals are not available (for some reason such as multipath reception, GPS jamming and spoofing, system failures, diving underwater, moving underground, etc.), the GMC will automatically switch from the GPS mode to the holdover mode, and output the stand-alone OCXO signals (OCXO pulses). The event-counter (EC) unit counts the GMC signals at a rate of *N* Hz. At the same time, the GMC signals are fed to the TDC as the start signal. The muPS signals (e.g. photomultiplier tube (PMT) outputs) are discriminated with the constant fraction discriminator (CFD) and fed to the TDC as the stop signal so that the time difference between the muPS signal and the GMC signal is measured (Fig. 2A). If the muPS signals are not fed into the TDC before the next GMC signal, the previous start signal is discarded. The EC and TDC are equipped with the reference detectors and the receiver detectors; here they are respectively labeled as the reference EC/TDC and the receiver EC/TDC. The reference TDC is a four-channel multi-stop TDC which receives the signals from four reference detectors. Also, the TDC time range is sufficiently longer than *N*^−1^ (in the current case *N*^−1^ = 100 ns).

The muon’s time of flight (TOF) measurement process is as follows. Initially, the reference EC and the receiver EC are located close to each other in order to reset by sending a reference signal. Subsequent to resetting, the reference EC and the receiver EC starts to count the number of OCXO pulses. By following this procedure, these two ECs are initially synchronized but this synchronization will gradually deviate according to the OCXO’s frequency drift level. When the muPS signals are fed into the TDCs, the numbers of EC counts (*n*_REFERENCE_ and *n*_RECEIVER_) are outputted from each EC. Therefore, the time (*t*_REFERENCE_ and *t*_RECEIVER_) when muPS signals are fed into each detector can be derived by:

6-1 $$ t_{{{\text{REFERENCE}}}} = n \times N_{{{\text{REFERENCE}}}}^{{ - {1}}} + \delta t, $$

6-2 $$ t_{{{\text{RECEIVER}}}} = n \times N_{{{\text{RECEIVER}}}}^{{ - {1}}} + \delta t, $$

where *δt* is the time difference between the start and stop signals fed into the TDCs. Due to the OCXO’s frequency drift generally *N*_REFERENCE_^−1^ ≠ *N*_RECEIVER_^−1^. The date on *t*_REFERENCE_ and *t*_RECEIVER_ are transferred to the local computer via Wi-Fi or an acoustic modem to calculate the TOF (*t*_TOF_):

7 $$ t_{{{\text{TOF}}}} = t_{{{\text{RECEIVER}}}} - t_{{{\text{REFERENCE}}}} $$

Figure 2B shows the time series of the logic levels output from the GMC (top) and those outputs from the TDC (bottom). According to Tanaka (2020)^19^, since the jitter level associated with *δt* is less than 200 ps, fluctuations in *N*^−1^ are a major factor to degrade the current TOF measurement quality. The errors in *N*^−1^ will be described in the following subsections.

### Grandmaster clock stability

**Figure 3:**
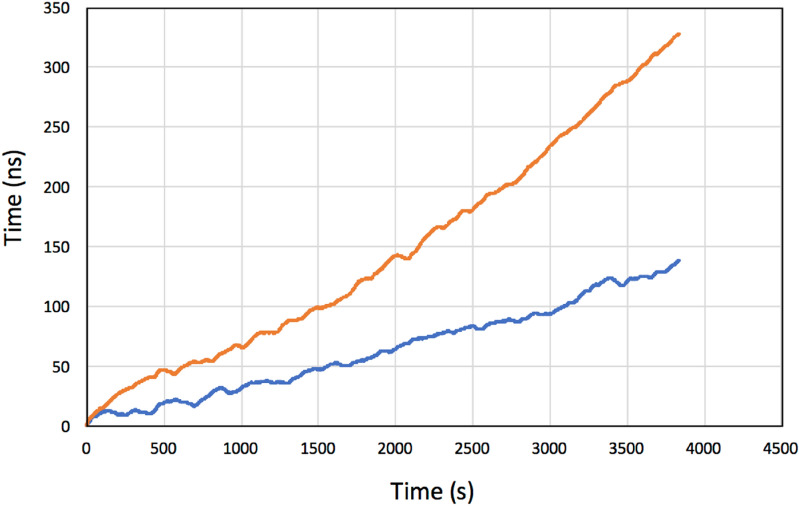
Averaged current OCXO time offset (<Δ*t*>) (blue lines) and the standard deviation (SD) of σ(*t*) (orange lines) as a function of time.

The Grandmaster clock (GMC) stability is the key component to define the accuracy of MuWNS. Experimental results of the current GMC/holdover are described here. The timing jitter measured with the current GMC is shown in Fig. 3. These time-dependent variation plots were drawn in the following way. The OCXO used in the current work (Trimble OCXO) was equipped inside the GPS grandmaster clock (Trimble Thunderbolt PTP GM200). In this study, the Trimble OCXO was initially synchronized with the GPS signal by connecting a GPS antenna (See “Method” section). The OCXO was then subsequently disconnected to simulate indoor/underground/underwater navigation. This process was repeated 12 times to investigate the drift pattern. The results showed that the drift speed varied in each run, and both positive and negative drifts were measured. If the OCXO operation time lasted less than 1 h, the drift was roughly linear as a function of time, but during longer operations, the behavior was unpredictable. Figure 3 shows the averaged time offset (the deviation between the GPS and holdover mode averaged over the entire run: <Δ*t* (*t*)>) and the standard deviation (SD) of σ(*t*) as a function of time. As can be seen in Fig. 3, <Δ*t* (*t*)> and σ(*t*) increase linearly as time goes by, and those fitted with a linear function are respectively:

8 $$ < \Delta t\left( t \right) > \left[ {{\text{ns}}} \right] = 0.0{534}t\left[ {\text{s}} \right] - {12}.{99},\,{\text{and}} $$

9 $$ \sigma \left( t \right)\left[ {{\text{ns}}} \right] = 0.0{81}t\left[ {\text{s}} \right] - {1}0.0{29}. $$

Equation (9) indicates that it is expected that the receiver time will be deviated from the reference time at a rate of ~ 8 ns every 100 s.

Since both the drift speed and the drift direction were unpredictable, σ(*t*) will remain as a non-zero factor. In MuWNS operations, Δ*t* can be derived by solving Eq. (2), but non-zero σ will lead to a major positioning error.

### Time synchronization between reference clocks and underground/underwater clocks

In order to evaluate the time synchronization capability in Eq. (3) between the reference and receiver detectors with the currently designed MuWNS, the relationship between the GMC stability and the muon tracking frequency were studied. If the muon tracking frequency was too low, the uncertainties in the drift level and direction would increase, and σ(*t*) would not be negligible, and as a consequence, quartic equations in Eq. (3) cannot be approximated as simultaneous equations anymore.

### Required spatial density of the reference detector network and resultant navigation performance

In order to evaluate spatial density of reference detectors required for achieving a given level of navigation performance of the currently designed MuWNS, the positioning accuracy and the time required for attaining this accuracy were studied. Generally, in the scheme of muPS, recording more muon tracks will improve the positioning accuracy more since the muon's arrival positions to the detector would be better averaged over the detection area, thus more muon events provide better positioning precision^19^. In the MuWNS scheme, the positioning errors coming from SD of Δ*t* have to be considered in addition to the positioning errors coming from this detector size effect. Since σ increases as time goes by, a tradeoff must be made between the muon's tracking frequency and the positioning accuracy. In the following discussion, we assume the detector size is negligible in comparison to an error Δ*L*_*i*_, where:

10 $$ \Delta L_{i} = c_{0} \sigma \left( t \right). $$

From Eqs. (9) and (10), the error in determining *L*_*i*_ in Eq. (2) increases at a rate of 2.4 m every 100 s. Ideally, if we can collect all of the muon tracks that pass all of the reference and receiver detectors (four tracks in total) at once, σ(*t*) will be the same for four equations and thus, such a time offset can be canceled out by solving Eq. (3). However, Eq. (4) indicates that we need a certain minimum time duration to collect these muons; hence σ(*t*) is not generally the same. Therefore, it is important to estimate the time required to collect sufficient number of muon events for navigation. A general description of this effect will be given below, but several case studies for this will be discussed later in the “Discussion” section.

**Figure 4:**
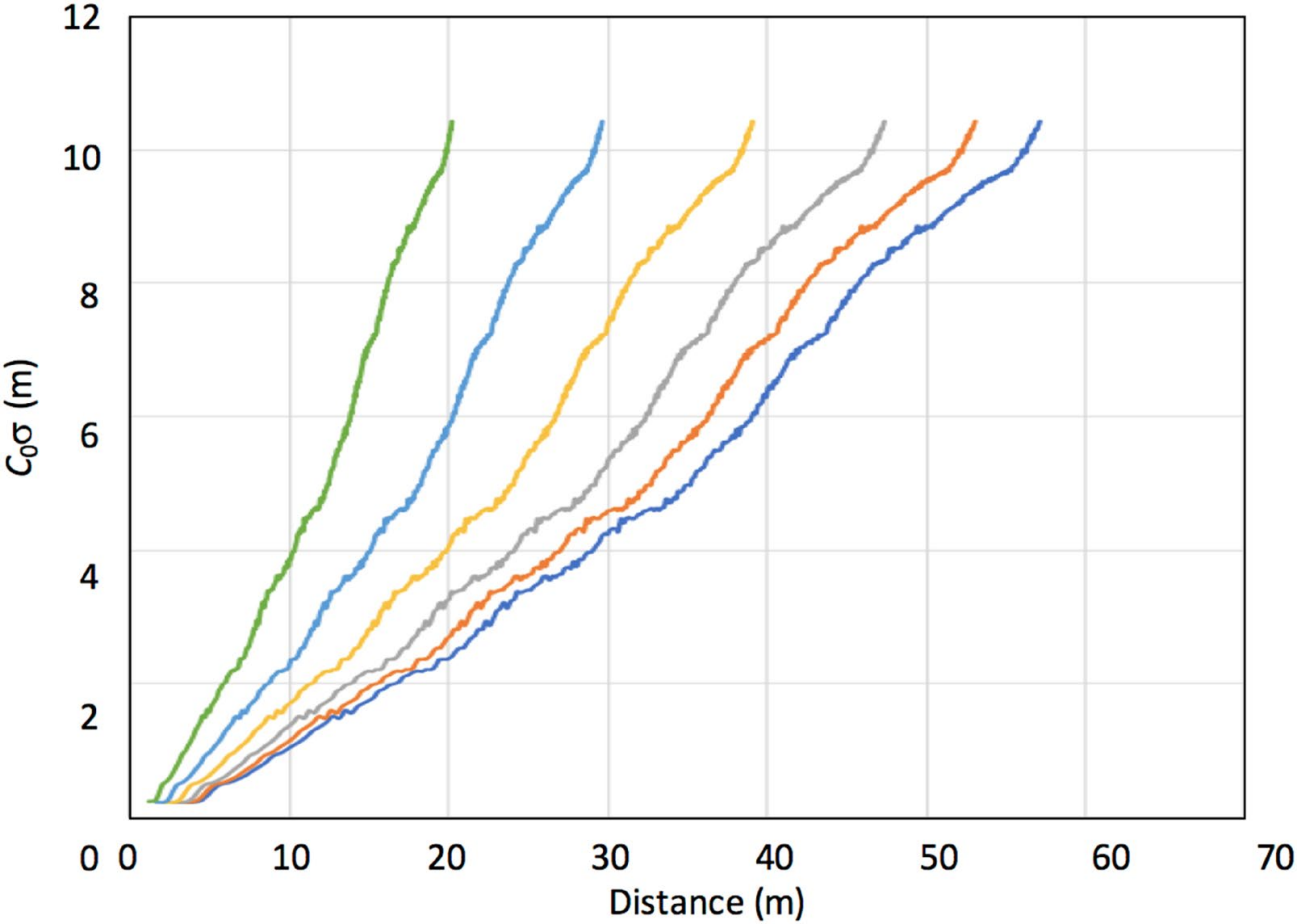
Measurement accuracy (*c*_0_σ(*t*)) as a function of the maximum distance between the reference and receiver detectors for various muon arrival angles from horizon (green: 100 mrad; sky blue: 150 mrad; yellow: 200 mrad; gray: 250 mrad; red: 300 mrad; and blue: 350 mrad).

Figure 4 shows *c*_0_σ(*t*) as a function of the maximum distance (*D*_MAX_(*θ*)) between the reference and receiver detectors, where *θ* is an elevation angle, and the maximum distance was defined as the distance (i.e., *SΩ*) that enables each pair of the reference and receiver detectors to record at least one muon track within the given time so that *c*_0_σ(*t*) is less than the given accuracy level. In other words, if the distance between the reference and the receiver detectors exceeds this maximum distance, the given positioning accuracy will not be attainable due to the limited flux of cosmic-ray muons. Therefore, the maximum distance defines the required density of the receiver detectors for the required positioning precision. In this calculation, there is generally less flexibility to increase the detector size, the effective active area of the reference and receiver detectors were both fixed to be 1 m^2^, and a typical basement floor without surface structures above it was assumed; namely, the assumed characteristics were: 5-m thick soil (SiO_2_) with an average density of 2.0 gcm^−3^ existing between the reference and the receiver detectors. As the muon's arrival angles approach the horizontal angle, the flux is reduced.

Coincidence events within the time window narrower than 1 µs identify whether the same muons are detected by both the reference detector and the receiver detector. Considering the vertical muon rate at 70 m^−2^ sr^−1^ s^−1^, this time window width allows accidental coincidence events every 4 h, that is much longer time scale than that we are discussing here. As can be seen in Fig. 4, *D*_MAX_ and *c*_0_σ(*t*) has a roughly linear relationship, and the fitted results are:

11-1 $$ D_{{{\text{MAX}}}} \left( {{35}0\,{\text{mrad}}} \right) = {4}.{81}c_{0} \sigma \left( t \right) + {8}.{41}, $$

11-2 $$ D_{{{\text{MAX}}}} \left( {{3}00\,{\text{mrad}}} \right) = {4}.{46}c_{0} \sigma \left( t \right) + {7}.{8}0, $$

11-3 $$ D_{{{\text{MAX}}}} \left( {{25}0\,{\text{mrad}}} \right) = {3}.{99}c_{0} \sigma \left( t \right) + {6}.{98}, $$

11-4 $$ D_{{{\text{MAX}}}} \left( {{2}00\,{\text{mrad}}} \right) = {3}.{29}c_{0} \sigma \left( t \right) + {5}.{75}, $$

11-5 $$ D_{{{\text{MAX}}}} \left( {{15}0\,{\text{mrad}}} \right) = {2}.{49}c_{0} \sigma \left( t \right) + {4}.{36}, $$

11-6 $$ D_{{{\text{MAX}}}} \left( {{1}00\,{\text{mrad}}} \right) = {1}.{7}0c_{0} \sigma \left( t \right) + {2}.{98}, $$

This corresponds to where the elevation angle values in the parenthesis are the angles between the reference-receiver lines and horizon. Generally, *D*_MAX_ has to be shorter to achieve a given navigation accuracy when the available muon incident angles are near horizontal. This is because the muon flux is reduced as its arriving angle approaches to the horizon; hence *D* in Eq. (5) must be shorter to attain larger *Ω* since this speeds up the process of collecting four muon tracks. In this case, it is necessary to place reference detectors more densely. Consequently, if the angles between the reference-receiver lines and horizon is lower than 100 mrad, muPS is unpractical. The placement position of the reference and receiver detectors and geological/topographical factors affect the time required for collecting muons; hence the positioning accuracy. This issue will be discussed further in the “Discussion” section.

**Figure 5:**
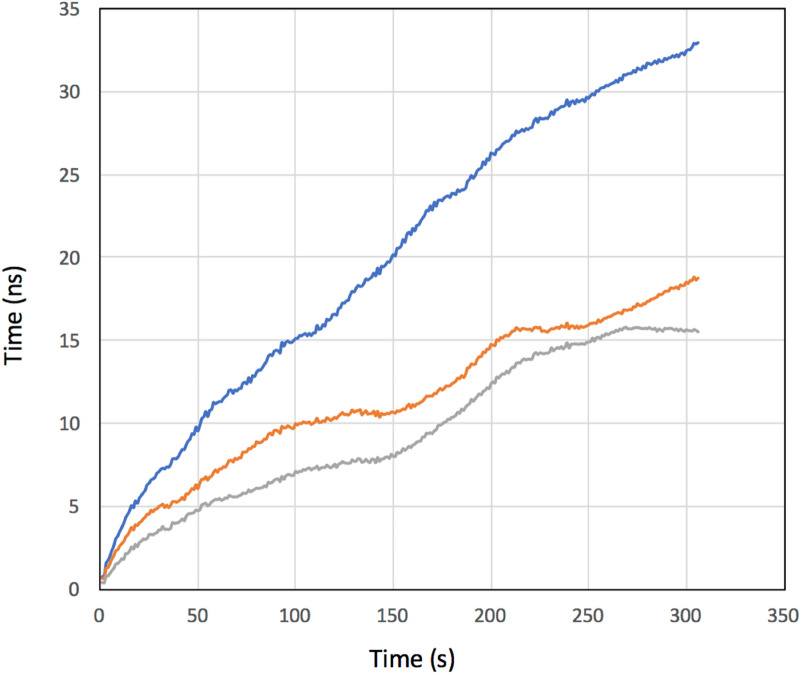
Time-dependent standard deviation (SD) of Δ*t* (σ(*t*)) averaged over single (blue lines), double (orange lines) and triple (gray lines) OCXO outputs.

Although the OCXO's frequency drift depends on a complicated combination of the environmental factors, it was expected that the resultant σ(*t*) would be suppressed if we utilize multiple independent OCXOs and take an average of σ(*t*) between them. Figure 5 compares σ(*t*) averaged over single, double and triple OCXO outputs. For example, if we use three OCXOs, the time we are allowed to keep the low drift level to attain VPL will be extended from less than 9 s to less than 28 s. As a result, a less dense reference detector array will be required for a multiple OCXO system.

**Figure 6:**
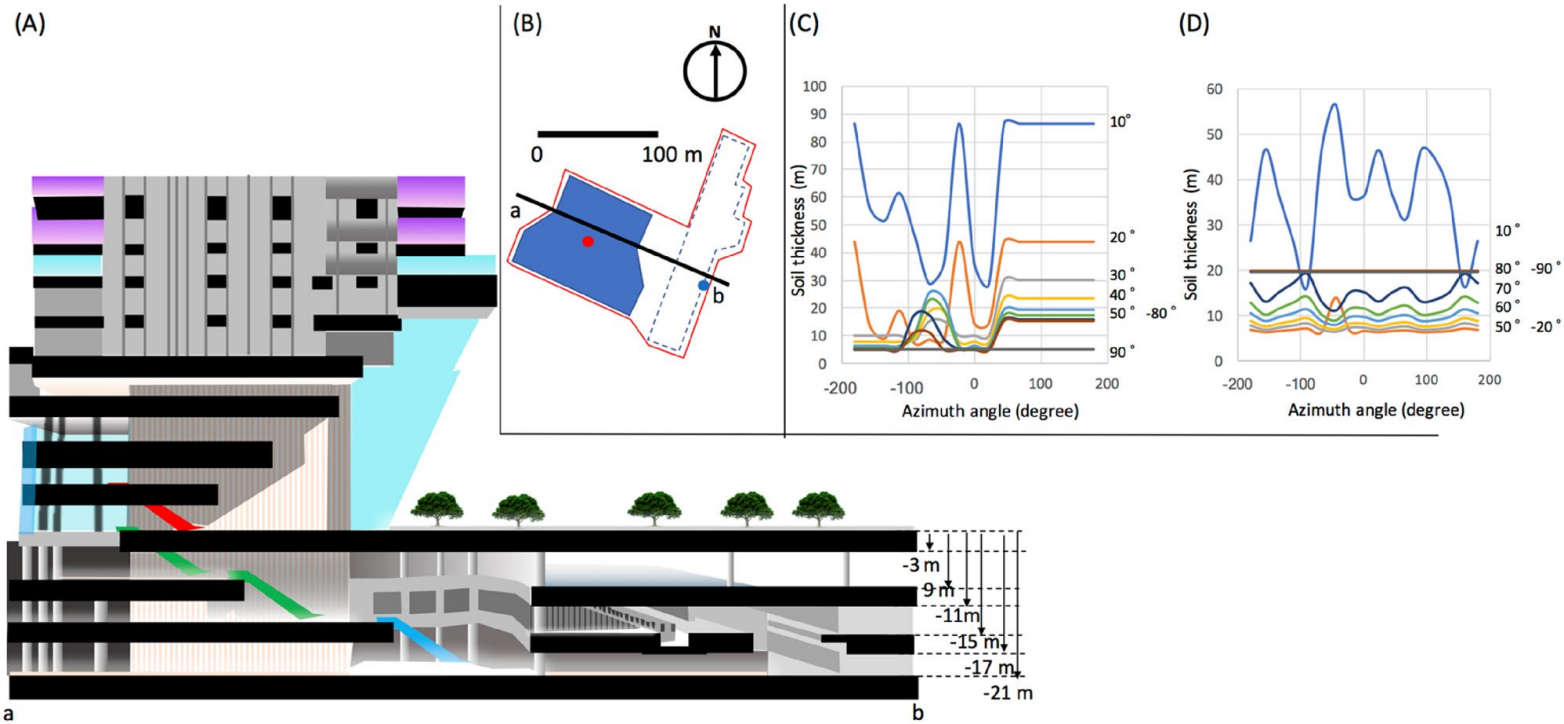
Schematic view of the Toranomon Hills Station Tower (THST) complex (**A**). The top view of the complex is shown in (**B**). The a-b line indicates the intersectional line of the cross-sectional view shown in (**A**). The red lines indicate the underground space of the complex. The red and blue filled circles respectively indicate the position of "A" and "B" used for the discussion in the main text. The corresponding soil/concrete thickness distributions are respectively shown in (**C**,**D**). The copyright of this image is owned by HKMT.

## Discussion

In this section, we will investigate possible MuWNS deployment scenarios in actual underground and undersea environments as example model cases. In the following case studies, the time required to go to the underground and underwater environments was neglected for the sake of simplicity.

### Shallow underground

The geometrical configuration of the first example case is shown in Fig. 6. In this case, the Toranomon Hills Station Tower (THST) complex, a structure that will soon be built integrally with the subway station (Toranomon Hills Station) on the Tokyo Metro Hibiya line, was chosen as an example. THST was designed as a 51-story (49 above ground floors and 2 basement floors) building that accommodates a hotel and offices that directly connects to the subway station via an underground shopping center. For the current evaluation, two locations were chosen in this complex: (A) right underneath the THST and (B) at the Toranomon Hills Station (Fig. 6A,B). The underground space and the THST were modeled using empty voxel finite elements (0.0 gcm^−3^) each measuring 10 × 10x10 m^3^. The area surrounded by the red lines is used for the underground space, and the size of this area measures 14,300 m^2^. In the THST region, it was assumed that the floor slabs with a thickness and density respectively of 30 cm and 2.0 gcm^−3^ were vertically spaced every 4.4 m. The density of the soil/basement floors was also assumed to be 2.0 gcm^−3^. The thicknesses of the basement floors match the values shown in Fig. 6A, and the receiver was assumed to be located on B2F (at a depth of 15 m).

Figure 6C,D shows the azimuthal distribution of the soil/concrete thickness at Locations A and B. Location A is located right underneath the skyscraper. At Location A, by integrating Eq. (3) over the entire azimuthal range for different elevation angles, we found that this skyscraper effect was only effective above elevation angles larger than 40° (ranging 10%-70% of the open-sky flux for 40°–90° from horizon). Below 40°, the soil thickness is as much as 10 m. At Location B, the THST effect was even weaker since only the westward component of the muon flux was affected by this building. Instead, the eastward component of the muon flux was more affected by the solid soil. Below 40° at Location B, the soil thickness was more than 40 m and thus, the muon flux arriving from this direction was reduced by 50%. Since the soil/concrete thickness was less than 10 m in the angular region below 40°, except for the edge of the underground space (red lines in Fig. 6B), the relationship shown in Fig. 4 can be directly applicable to this case. From Fig. 4, since the maximum distances between the reference and receiver detectors (*D*_MAX_) allowed for attaining accuracies less than 3 m is 25 m for the elevation angular region above 300 mrad (17°), the interval between the reference detectors must be shorter than 2 × (*D*_MAX_^2^ − *depth*^2^)^1/2^ = 2 × (25^2^[m^2^] − 9^2^[m^2^])^1/2^ = 46 m. In conclusion, in order to attain PL, the reference detectors had to be located at an interval of 46 m. These spatial densities respectively require 7 reference detectors to cover the current complex area of 14,300 m^2^ (14,300[m^2^]/46^2^[m^2^] = 6.75). The cost required for developing MuWNS in this case will be discussed later. With this geometrical configuration, the times required to attain VPL and PL are respectively 28 s and 176 s. Here in this discussion MuWNS with triple OCXO (Fig. 5) was assumed. These time resolutions improve in proportion to the aperture size of the reference or receiver detectors. While it would be unrealistic for a pedestrian to carry a 1 m^2^ detector as a handheld navigation system, the purpose of the current discussion is to model MuWNS operations in shallow underground/indoor environments. To adapt this system for handheld pedestrian navigation devices, several receiver detectors fixed to the basement floor could be combined with Wi-Fi or Bluetooth to view on smartphones.

### Deep underground

**Figure 7:**
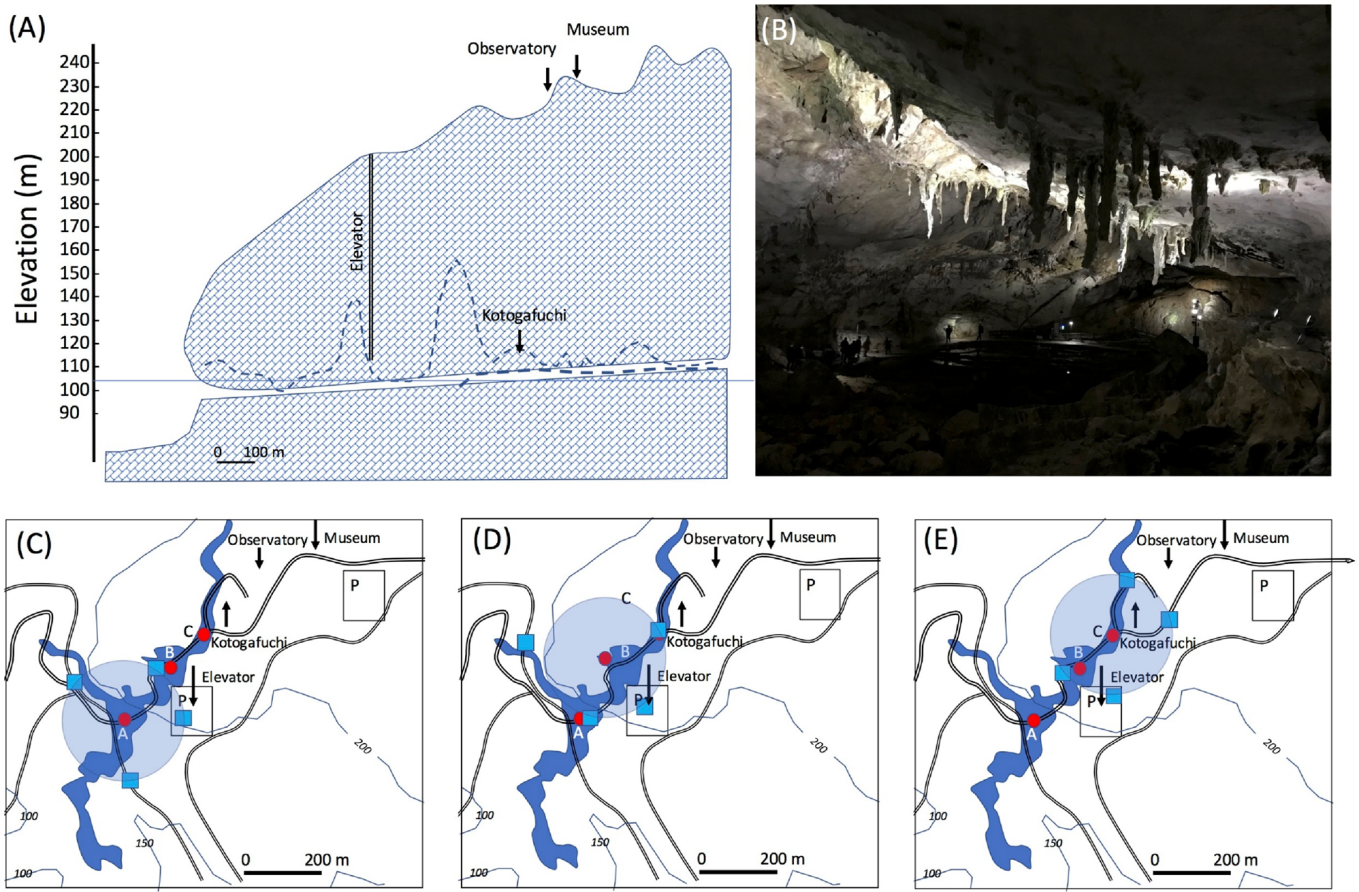
Side (**A**), inside photograph (**B**) and top views (**C**,**D**,**E**) of Akiyoshido cave. The dashed line indicates the actual geometry of the cave, but a cylindrical cave (empty near horizontal space underneath the overburden) was assumed in this work (**A**). In the top views, the locations of the reference detectors (blue filled squares) and receiver detectors (red filled squares) are shown. The cave region is indicated by the region filled in with darker blue. The locations of the reference detectors (blue filled squares) and receiver detectors (red filled squares) are also shown. Blue filled circles indicate the region within 150 m from the receiver detector. The double lines and the labels "P" respectively indicate roads and parking lots. The contour lines showing elevations are also shown. HKMT took the photograph. HKMT drew the map and the images with Microsoft PowerPoint software and holds the copyright.

The geometrical configuration of the second example case is shown in Fig. 7. In this case, Akiyoshido cave, Japan was chosen as an example. Akiyoshido cave is a limestone cave located underneath Akiyoshidai plateau, one of the largest karst plateaus in Japan, stretching over a total area of 54 square kilometers. The cave extends around 10 km lengthwise with ceilings reaching up to 80 m high. The tourist route is limited to regions approximately in a 1 km radius from the entrance. The Akiyoshidai area is well developed as a tourist's site; roads, cafes, museums, and observatories have been constructed above the cave. Therefore, it was relatively simple to design the experiment with realistic logistics.

**Figure 8:**
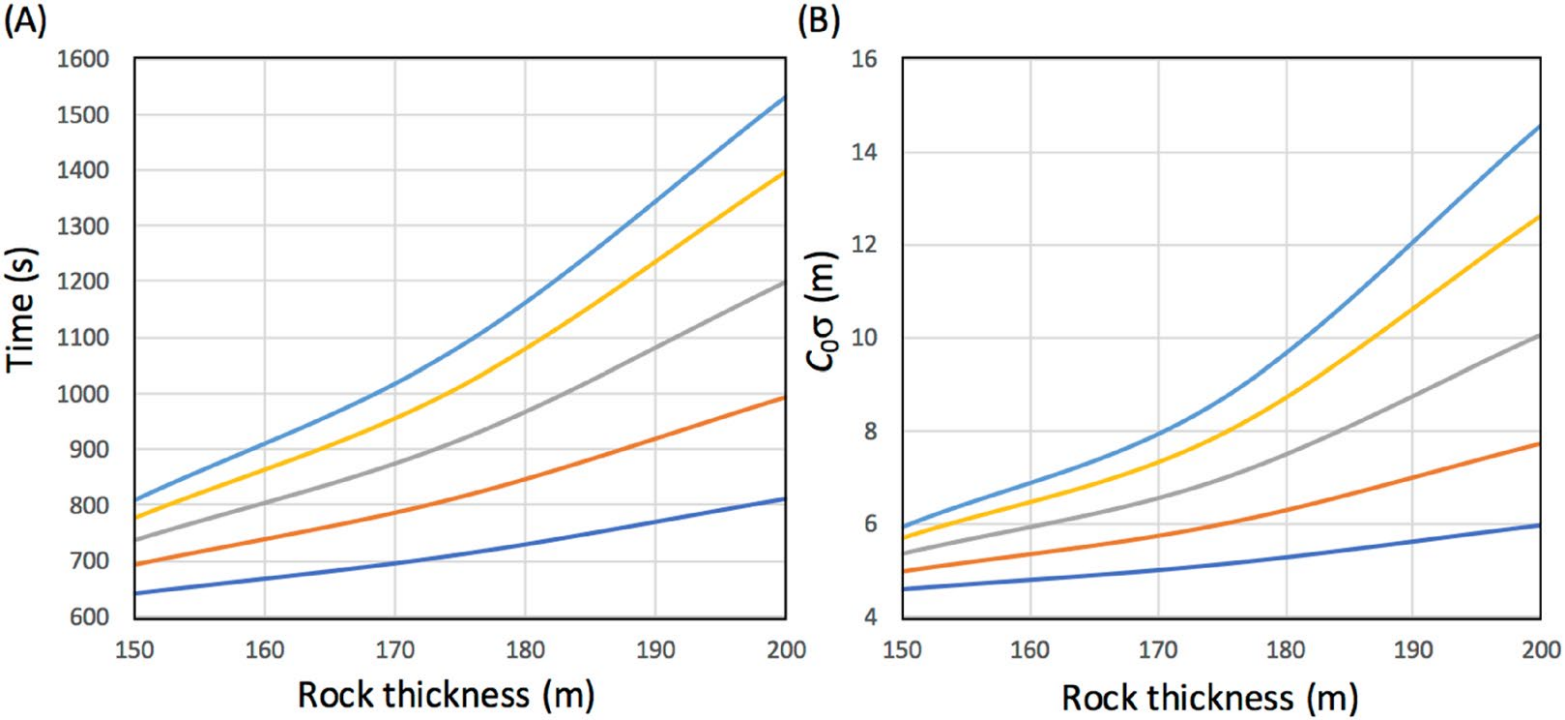
Time required for recording one muon track as a function of rock thickness for various muon arrival angles from horizon (from top to bottom—sky blue: 500 mrad; yellow: 450 mrad; gray: 400 mrad; red: 350 mrad; and blue: 300 mrad) (**A**) and the corresponding *c*_0_σ (**B**).

As can be seen in Fig. 7, since the rock thickness situated between the reference and receiver detectors was much thicker than the first case (shown in Fig. 6), the size of the detector had to be larger. However, since it is unrealistic to enlarge the reference detector, it was assumed that a much larger reference detector with a detection area of 12 × 2.5 m^2^ was hypothetically installed in a 40-foot (12 m) standard freight container. Container trailers as large as 53 feet (15.9 m) could be rented, but since this 53-foot size is one of the largest sizes categorized for modern shipping, we have instead chosen a more common 40-foot container size since this is smaller and more convenient for standard truck-borne applications. In this scheme, the reference detectors are mobile. In other words, the number of receiver detectors can be reduced if the total operation period doesn't exceed the time required for attaining a given positioning precision level since these can be moved. Figure 7B–D show the possible positions of the tracks on the road or parking lots located above the cave. In this geometry, the overburden rock thicknesses range between 150 and 200 m, thus the viewing angle of the receiver detector was more or less than 1 msr, and the elevation angle between the receiver and reference detectors ranged between 300 and 500 mrad. Figure 8A shows the time required for recording one muon track as a function of rock thickness for various muon arrival angles from horizon. The number of reference detectors and the size of the receiver detector were respectively assumed to be four and 1.7 × 1.7 m^2^. Figure 8B shows the corresponding *c*_0_σ. In this case, it was found that the current MuWNS setup was sufficient to achieve a UL level of navigation. The rate of the muon that passes both the reference and the receiver detector depends on the thickness of the rock. As shown in Fig. 8B, this rate affects the positioning accuracy, but since the thickness of the rock overburden can be derived by measuring the muon rate in a muographic way, this positioning accuracy can be estimated when performing muPS measurements. It is worth mentioning that the time required for muons to travel from the ground surface to underground regions doesn’t change since the speed of the muons is always the speed of light in a vacuum even when traveling through rock.

The digital Through-The-Earth (TTE) communication system can be used to provide communications links from the surface to below-ground locations. The system can be used to provide real-time monitoring of equipment sensors. The TTE communication uses very low frequency (VLF) transmission (transmission is usually done with magnetic induction, at frequencies below 30 kHz) to provide reliable data links through environments surrounded by rock, but this system in turn severely limits the bandwidth available for information transmission with data rates ranging from 9 bps to 1 kpbs^43^. However, since both reference and receiver detectors have their own clock, the information that would have to be transferred to the counterpart detector to be effective is just *n* and *t*. Therefore, this data rate is sufficient for MuWNS communication requirements.

### Undersea navigation

**Figure 9:**
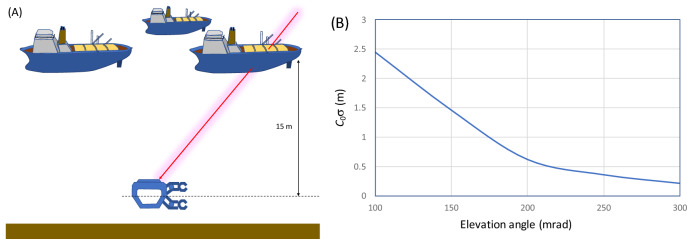
Conceptual view of the undersea MuWNS in conjunction with the Robotic Vessels as-a-Service (RoboVaaS) (**A**). Yellow panels on the Unmanned Surface Vehicles (USVs) indicate the reference detectors to navigate an Unmanned Underwater Vehicle (UUV). The red line indicates the muon trajectory. The *c*_0_σ as a function of the muon arrival angles from the horizon is also shown (**B**). The copyright of this image is owned by HKMT.

In the previous work, the application of wired muPS to positioning of the seafloor was discussed^19^. There, the usage of a GPS buoy was modeled. One reference detector is attached to the buoy, and the natural whirling motion of the buoy collects the data for different locations above the receiver detector that is attached to the anchor of the buoy. The signal and power cables used to connect the reference and receiver are arranged along with the wire connecting the buoy and the anchor. In this work, an application of more flexible wireless muPS is discussed. The geometrical configuration of the third example case is shown in Fig. 9A. In this case, a shallow bay (< 20 m) such as Tokyo Bay was chosen as an example, and a new scheme called the Robotic Vessels as-a-Service (RoboVaaS) was considered. RoboVaaS intends to revolutionize shipping and near-shore operations in coastal waters by integrating and networking a smaller Unmanned Surface Vehicle (USV) and an Unmanned Underwater Vehicle (UUV) efficiently in order to offer new services for shipping^44^. In this scheme, it was assumed that a number of USVs that carry reference detectors would navigate a number of UUVs operated at a depth of 15 m below sea level. The total size of the active area of the reference detector was assumed to be 20 × 5 m^2^, a size which could be easily accommodated on a small cargo boat. The size of the receiver detector was assumed to be 1.0 × 1.0 m^2^. It was also assumed that the positions of reference detectors can be defined in real time. Figure 9B shows the *c*_0_σ as a function of the muon arrival angles from the horizon. If we use more slanted muons, higher muon flux is available; hence the value of *c*_0_σ would be smaller for the given detector size and *Ω*, but a denser reference detector network would be needed. In order to plot this graph, *E*_c_ in Eq. (3) was derived from the CSDA muon range^39^ for liquid H_2_O with a density of 1.0 gcm^−3^, and used for the lower limit of the integration process. The derived integrated flux was used for calculating the time required to record at least one muon track that passed through both a reference and a receiver detector. This time was converted to *c*_0_σ by using the *t*-*σ* relationship shown in Fig. 5. As a consequence, it was found that the muon's arrival angle could be as low as 100 mrad and 175 mrad to achieve PL and VPL. These angles determine the USV network density required for attaining PL and VPL: 44 km^−2^ and 135 km^−2^.

### MuWNS time synchronizer

Fifth-generation (5G) mobile/cellular radio access network (RAN)^45^, industrial automation and control systems^46^, as well as land^47^ and ocean^48^ observation networks all require real-time connectivity with precise time synchronization to provide robust reference time information to the devices located in these networks on a common time basis with a jitter level below 1 microsecond^45^. Such requirements are typically fulfilled through wired technologies like Time-sensitive Networking (TSN)^49^. However, wireless technologies offer various benefits for network communication^50,51^. MuWNS also offers a unique wireless time synchronization capability without GPS signal input, and the expected jitter level is less than 3.3 ns and less than 10 ns respectively for the VPL and PL mode. This means that if the reference detectors are receiving the GPS signals, the receiver detectors could get the absolute time in indoor, underground, or underwater environments.

### Detector and operational costs

The MuWNS operational cost is very low. In the first case (Fig. 6), the required detectors are six 1-m^2^ reference detectors and one 1-m^2^ receiver detector for PL navigation. The cost of each 1-m^2^ plastic scintillator and photomultiplier tube (PMT) are respectively 5000 USD and 1000 USD. Each GMC and TDC costs 4000 USD. Therefore, the cost required for constructing 6 reference stations will be 84,000 USD. In the second case (Fig. 7), considering the required positioning resolution we need and the attenuation length of the scintillator of ~ 4 m, 6 scintillator panels, each measuring 2.5 × 2-m^2^ would be used to make 30-m^2^ detection area. Applying the same unit cost (5000 USDm^−2^), the plastic scintillator cost would be 150,000 USD. The number of PMTs, GMCs, and TDCs is the same as the first case. Therefore, the cost required for constructing the reference station will be 204,000 USD. In the third case (Fig. 9), 16 scintillator panels, each measuring 2.5 × 2.5-m^2^ would be used to make a 100-m^2^ detection area. Applying the same unit cost (5000 USDm^−2^), the plastic scintillator cost would be 500,000 USD. The number of PMTs, GMCs, and TDCs required for constructing the reference station would increase to 16 each. Therefore, the cost required for constructing the reference station will be 644,000 USD. The cost required for preparing the receiver detector is usually much smaller than that required for constructing the reference stations. These costs are 15,000 USD, 24,000 USD, and 15,000 USD in the first, second and third cases, respectively. In the third case a pressure vessel that costs 7000 USD will additionally be needed. In the above cost estimates, the costs required for any logistics, for the Wi-Fi/VLF/acoustic communication system, and other accessories required for the outdoor measurements are not included. Even though the first investment costs are high in some cases, since MuWNS is a passive navigation system, the power consumption is low; thus, the operational costs would be much lower in comparison to other active navigation systems.

A comparison between the wired muPS described in the prior work^19^ and the current work is summarized in Table 1. There are two distinctive differences between these techniques which relate to the following 2 issues: (A) the time synchronization accuracy and (B) the time required for positioning. A picosecond level synchronization accuracy can be achieved with the wired technique. Thus, wired muPS has no time-dependent positioning drift and thus, centimeter level precise positioning measurements are possible with sufficiently long (timescale) measurement periods. However, since the reference detectors and the receiver detector are connected with a physical cable, the mobility of the receiver detectors is restricted; hence wired muPS generally cannot be used for the purpose of navigation. On the contrary, while the wireless technique can be applied to mobile experiments, due to unexpected frequency drift coming from the local clock, the positioning accuracy is degraded. The resultant positioning accuracy depends on the frequency of cosmic-ray muon arrival to the detectors, which is defined by Eq. (3). Since we cannot change the muon flux, we need to use more stable clock to improve this situation. The GMC with a Cs-oscillator-based holdover guarantees an order of magnitude higher stability than the OCXO-based holdover. However, OCXOs, which cost a hundred to a few hundred US dollars (USD), are far less expensive than atomic clocks that cost hundreds of thousand dollars. The current study clarified that the MuWNS can operate with realistic logistics at reasonable costs.

**Table 1:** Comparison between the wired muPS^19^ and the current work.

	Wired muPS^19^	MuWNS
Time synchronization accuracy (ns)	< 0.027	3–30
Positioning accuracy (m)	0.02–0.1	1–10
Time required for positioning (s)	~ 10^7^	20–1500

In the current work, in order to remove the restriction on receiver detector mobility that existed in the previous work^19^, the GMC-holdover-based MuWNS was designed and evaluated its performance based on the GMC-OCXO data. Although the x–y-z positioning accuracy strongly depends on the geometric configuration of the reference and receiver detectors, we could conclude that the current MuWNS functions at a useful depth level in the shallow underground/indoor, deep underground and underwater. In conclusion, based on the results of the current experiments and the simulations, it has been shown that a navigation accuracy of $\lesssim$ 3 m, $\lesssim$ 10 m and  $\lesssim$ 1 m can be respectively achieved in shallow underground, deep underground, and underwater environments at reasonable costs. It is anticipated that MuWNS has the potential to become a practical and well-used new navigation system that could be applied to a variety of underground and underwater environments in the humanosphere (the regions of Earth utilized by and explored by humans) as a sustainable navigation system that doesn't require any active navigation signal generation.

## Method

### Experimental setup

In this demonstrational work, two independent GMCs (Trimble Thunderbolt PTP GM200) were used for evaluating the time synchronization capability between the aboveground/sea surface detectors and underground/underwater detectors. The GMC used for the time synchronization of the aboveground/sea surface detectors (aboveground GMC) was constantly connected to a GPS antenna to receive the time synchronization signal. The GMC used for the time synchronization of the underground/underwater detectors (underground GMC) was initially connected to a GPS antenna but then eventually disconnected. The GMC's PPS (pulse per second) signals were fed into the start and stop channels of a long-time range (10 microseconds) TDC (Sciosence TDC-GPX) with a time resolution of 27 ps to measure the OCXO frequency drift as a time difference between the PPS pulses; these pulses were synchronized with the GPS clock and the PPS pulses that were generated by the OCXO without a frequency comparison with the GPS signals. Since both a positive (forward) and negative (delay) directional drift were expected, a delay circuit (600 ns) was inserted between the underground GMC and the TDC. The minimum receivable pulse width (10 ns) of the current TDC was sufficient for the measurement described here.

## Data Availability

The datasets used and/or analyzed during the current study available from the corresponding author on reasonable request.
